# Italian Validation of the 12-Item Multiple Sclerosis Walking Scale

**DOI:** 10.1155/2015/540828

**Published:** 2015-03-26

**Authors:** C. Solaro, E. Trabucco, A. Signori, M. Cella, M. Messmer Uccelli, G. Brichetto, P. Cavalla, M. Gironi, F. Patti, L. Prosperini

**Affiliations:** ^1^Neurology Unit, Head and Neck Department, ASL 3 “Genovese”, 16154 Genova, Italy; ^2^Department of Experimental Medicine, Section of Diagnostic Radiology, University of Genoa, 16126 Genova, Italy; ^3^Department of Health Sciences, Section of Biostatistics University of Genova, 16126 Genova, Italy; ^4^Italian Multiple Sclerosis Society, 16149 Genova, Italy; ^5^Scientific Research Area, Italian Multiple Sclerosis Foundation, 16149 Genova, Italy; ^6^Department of Neurosciences, AOU S. Giovanni Battista, University of Torino, 10124 Torino, Italy; ^7^INSPE, Neuroscience Division, IRCCS HSR, 20132 Milano, Italy; ^8^CAM, Polidiagnostic Center, 20900 Monza, Italy; ^9^Department DANA, “GF Ingrassia”, Neuroscience Section (Multiple Sclerosis Centre), University of Catania, 95124 Catania, Italy; ^10^MS Centre, Department of Neurology and Psychiatry, Sapienza University, 00185 Rome, Italy

## Abstract

*Objective*. Gait impairment is commonly in people with multiple sclerosis (MS). The 12-item MS walking scale (MSWS-12) assesses patients' measurement of walking quality. The aim of this study was to cross-culturally adapt and validate the MSWS-12 for the Italian population with MS. *Methods*. Six MS out-patient clinics across Italy enrolled subjects between June 2013 and December 2013. Construct validity of MSWS-12 was determined by examining correlations with the Italian version of the EDSS, the timed 25-foot walk (T25FW), and the Fatigue Severity Scale (FSS). *Results*. 321 MS subjects were enrolled. Mean age was 47.55 years and mean disease duration was 13.8 years. Mean EDSS score was 4.46. 185 subjects had a relapsing-remitting course, 92 were secondary progressive, 43 were primary progressive, and 1 had a clinically isolated syndrome. The mean total score of the MSWS-12 was 49.6 (SD: 31) with values ranging between 0 and 100. Correlations between the MSWS-12 with age, disease duration, and disease course were found but not with gender. Values of the MSWS-12/IT were significantly related to EDSS (0.71), to the T25FW (0.65), and to the FSS (0.51). *Conclusion*. MSWS-12/IT has been adapted and validated, it is a reliable and reproducible scale for Italian patients with MS.

## 1. Introduction

Multiple sclerosis (MS) is a chronic, autoimmune disease associated with progressive disability. The course of MS is heterogeneous, is generally diagnosed in adults between the ages of 20–50 years, and is characterized by multiple neurologic deficits and significantly decreased quality of life [1]. Gait impairment is reported as a primary complaint by 85% of people with MS and more than one-third are not able to walk 20 years after the diagnosis [2].

Walking impairment has often been measured in clinical research and practice using the Expanded Disability Status Scale (EDSS) [3], the timed 25-foot walk (T25FW) [4, 5], the 6-minute walk (6MW) [6, 7], and quantitative analysis (gait kinematics) [5].

The 12-item multiple sclerosis walking scale (MSWS-12) provides a patient-reported outcome of the impact of MS on walking [3]. A recent study confirmed the association between MSWS-12 scores and T25FW and 6MW performance and showed the association between MSWS-12 scores and spatial and temporal parameters of gait [8]. This provided evidence for the validity of the MSWS-12 as a measure of aspects of the quality of walking in addition to walking speed and endurance [8].

To our knowledge, the scale has only been translated into Portuguese for the Brazilian population [9].

The aims of the present study were to translate and adapt the MSWS-12 into Italian and to test the resulting Italian version in a group of patients followed in specialized MS outpatient clinics.

## 2. Methods

### 2.1. Subjects

The present study involved six MS outpatient clinics across Italy, between June 2013 and December 2013. Inclusion criteria were a confirmed MS diagnosis according to McDonald's [10], age greater than 18 years, ability to walk with or without the use of adaptive devices, and an EDSS range from 0 to 7. Patients experiencing an exacerbation within 30 days prior to the assessment, with an additional neurological disease or with one or more concomitant illnesses, were excluded from the study.

Ethical approval for this study was obtained from the ethical committees of each participating hospital. Signed informed consent was obtained from each patient prior to enrollment in the study according to the Declaration of Helsinki.

### 2.2. Translation and Adaptation

The MSWS-12 was translated into Italian by a professional translator with knowledge of health terminology. The translation was evaluated to ensure semantic equivalence and acceptability. During an initial meeting of MS experts, a list of possible alternatives for the controversial item stems and response choices was developed. Problematic items and response choices were retranslated into Italian from the original version and a definitive version was determined by consensus. Subsequently, the Italian version was back-translated into English and compared with the original one. The translated questionnaire was completed by 53 patients, the results of which were discussed at a second meeting that included patients and their proxies. Mean age was 46 and 56 (SD: 11.61, range: 18−67); 32 (60.37%) were female and 21 (39.63%) were male. Mean disease duration was 12 (SD: 5.5; range: 2–24), and mean EDSS score was 5.2 (range: 1.5–6.5). Most subjects had a relapsing-remitting (RR) course (*n* = 26; 49.1%), 15 (28.3%) were secondary progressive (SP), and 12 (22.6%) were primary progressive (PP); see Table 1.

Subsequently, the final version of the questionnaire (Table 2) was tested and retested after 15 days, on an additional group of 25 consecutively seen outpatients with MS at the coordinating center (56% woman, at age (mean ± SD) of 50 ± 11 years, and suffering from MS since 12 ± 5.5). See Table 1.

### 2.3. Administration of the Questionnaire

The MSWS-12/IT was explained to subjects by a neurologist. The scale includes 12 items scored on a 1 (not at all) to 5 (extremely) point scale. Scores on the 12 items are summed. To transform to a 0–100 scale, the minimum score of 12 is subtracted from the sum, and the result is divided by 48 and then multiplied by 100 [11–13].

The hypothesis is that MSWS-12/IT should correlate with objective measure of disability (EDSS), disease course, and walking speed (T25FW), while fatigue and multidimensional symptoms should be only incompletely related to MSWS-12/IT.

The EDSS assesses neurological impairment and disability due to MS in 20 steps from 0 (normal neurological examination) to 10 (death due to MS) [3].

The T25FW is a component of the Multiple Sclerosis Functional Composite (MSFC) [14] and measures the time needed to walk 25 feet (7.62 meters) with or without an assistive device as quickly and safely as possible. The final result is the mean time (in seconds) needed to complete two consecutive attempts of the T25FW.

The FSS is commonly used in MS. The subject is asked to read each statement and circle a number from 1 (strong disagreement) to 7 (strong agreement), depending on how appropriate they feel the statement applies to them over the preceding week [15].

Construct validity of MSWS-12 was determined by examining correlations with the Italian version of the EDSS, the timed 25-foot walk (T25FW), and the Fatigue Severity Scale (FSS).

## 3. Statistical Analysis

The means with standard deviations (SD) and ranges for the MSWS-12 and EDSS and the medians with ranges for the T25FW and FSS were calculated. Pearson correlation was calculated for MSWS-12 versus age and disease duration. Nonparametric Spearman coefficient was used for calculating the correlation between EDSS, FSS, T25FW, and MSWS-12. The MSWS-12 mean values from males and females were tested using Student's* t*-test for independent samples and an ANOVA was used with the same aim using disease course (4 groups) as the group indicator. Cronbach's alpha was calculated to assess the internal consistency of the MSWS-12 and intraclass correlation coefficient (ICC), using a two-way mixed effects model and correlation coefficient used to assess the test-retest performance of the scale after 15 days. Standard error of measurement (SEM) was also calculated to quantify error in the test-retest. The formula used for the SEM was (SD)^∗^. SEM, in a longitudinal setting, represents the intraindividual variability of the score. When the MS-12 score of a patient, after treatment, exceeds the baseline value of this quantity, evidence of a treatment effect is reasonable. A *P* value lower than 0.05 was considered statistically significant. SPSS (v.20; IBM Corp.) was used for the computations.

## 4. Results

321 patients were enrolled in the study between June 2013 and December 2013. Mean age was 47.55 years (range: 18–76); 158 (49.2%) subjects were male and 163 (50.8%) were female. Mean disease duration was 13.8 years (range: 1–42 years), and mean EDSS score was 4.46 (range: 0–6.5). The majority of subjects had a relapsing-remitting form of MS (RR) (*n* = 185, 57.6%), 92 (28.7%) were secondary progressive (SP), 43 (13.4%) were primary progressive (PP), and 1 (0.3%) had a clinically isolated syndrome (CIS) (Table 1). Mean time to complete the questionnaire was 2:20 minutes (range: 54 seconds–4.16 minutes). More than 90% of subjects reported that the questionnaire was easy to understand. All questionnaires were included in the analysis.

The mean total score of the MSWS-12 was 49.6 (SD: 31) with values ranging between 0 and 100. The T25FW had a median score of 8.9 seconds with values ranging between 3.37 and 95.06 seconds. The FSS had a median score of 30.8 with values ranging between 1 and 63.

### 4.1. Validity

Correlations between the MSWS-12 with age and disease duration were, respectively, 0.32 (*P* < 0.001) and 0.19 (*P* < 0.001), with no significant differences (*P* = 0.30) between males (mean: 41.1; SD: 24.3) and females (mean: 38.2; SD: 25.2). Values of the MSWS-12/IT were significantly related to disease course (*P* < 0.001), specifically lower values for RR (mean: 38.2; SD: 29.5) compared with SP (mean: 65.9, SD: 24.6) and PP (mean: 62.7; SD: 27.7). Spearman correlations between MSWS-12 and EDSS were 0.71 (*P* < 0.001) and between the MSWS-12 and T25FW were 0.65 while between the MSWS-12 and FSS were 0.51 (*P* < 0.001). Correlation between the MSWS-12 and TW25 was similar for patients with an EDSS < 3.5 (rho = 0.50) versus those with an EDSS ≥ 3.5 (rho = 0.48).

### 4.2. Acceptability

The global score on 321 patients ranged from the minimum to the maximum of all possible values and did not show a ceiling or floor effect. There was a possible ceiling effect for item 2 (64.6% of subjects answered 5 = extreme limitation).

### 4.3. Internal Consistency and Test-Retest Reliability

For internal consistency, a Cronbach's alpha, for the global score, of 0.97 was calculated. A correlation coefficient of 0.74 (*P* < 0.001) between baseline and 15-day scores and an ICC of 0.85 (0.62–0.94) were detected. A SEM of 12, corresponding to an intraindividual variability on MS-12 score of 24.2%, was found.

## 5. Discussion 

Hobart et al. developed a patient-based measurement on walking impairment, a critical aspect of physical functioning that plays a key role in the health-related quality of life of people with MS [4].

The degree of walking impairment is an important outcome for monitoring disease progression in MS. Patients' perception of the impact of MS on walking is extremely relevant. The purpose of the study was to adapt and validate the MSWS-12 for use in Italian subjects with MS. The results provide evidence that the MSWS-12/IT is reliable and valid. In addition, greater than 90% of subjects reported that this instrument is simple to understand and complete, thus, making it a useful tool in clinical practice.

In this study, the MSWS-12/IT was compared with the EDSS, T25FW, and FSS. The correlation between the MSWS-12/IT and EDSS was positive (0.69). Similar to an earlier study, higher disability corresponded to a greater impact on walking [15]. This may be expected given that higher disability often results in increased loss of balance, muscle weakness, fatigue, cognitive impairment, fear of falling, spasticity, tremor, and visual impairment, all symptoms that influence walking [16, 17]. The correlation between the MSWS-12/IT and the T25FW suggests that the time taken to walk a specific distance is the limiting factor in the subject's perception of their walking impairment. In addition, the weaker correlation between the MSWS-12 and the FSS suggests that fatigue has some impact on walking ability, although a limited one.

Gait impairment is reported as a primary complaint by 85% of people with MS [2] and, in general, is prevalent in the chronic phase of the disease. This is supported by the fact that subjects with an RR disease course had a mean MSWS-12/IT of 38.2, significantly lower than subjects in a progressive phase of the disease (PP 62.7, SP 65.9).

The results from the present study show a statistically significant relationship of the MSWS-12/IT with both age and disease course, while there was no significant difference related to gender.

One limitation of this study is that mood and cognitive functions were not assessed. It could be suggested that mood disorders, such as anxiety or depression, may influence subjects' perception of the limitations of their disease on walking. Impairment of cognitive functions could influence one's ability to accurately complete the scale although the vast majority of subjects (90%) reported that the questions were clear and simple.

In conclusion, the MSWS-12/IT has been adapted and validated for Italian patients with MS and is comparable to the original version (MSWS-12) regarding semantic, cultural, and idiomatic characteristics.

The validated scale and scoring instructions are available in Italian free of charge by contacting the corresponding author.

## Figures and Tables

**Table 1:** Sample characteristics.

	Subjects who completed translated version (*n* = 53)	Subjects tested and retested (*n* = 25)	All subjects (*n* = 321)	Subsample with information on items (*n* = 82/321)	*P* value for difference
Age (years), mean (SD); range	46.56 (11.61); range: 18–67	50 (11); range 35–76	47.55 (11.9); range: 18–76	44.55 (10.2); range: 21–76	0.008
Gender: female/male	32 (60.37%)/21 (39.63%)	14 (56%)/11 (44%)	163 (50.8%)/158 (49.2%)	49 (59.8%)/33 (40.2%)	0.079
Disease duration (Years), mean (SD); range	14.9 (9.1); range: 1–42	12 (5.5); range 2–24	13.8 (8.3); range: 1–42	13.4 (6.4); range: 2–34	0.57
EDSS score, mean (range)	5.2; range: 1.5–6.5	5.5; range: 3–7	4.46; range: 0–6.5	4.46; range: 1–7	0.74
Relapsing-remitting course (RR), *n* (%)	26 (49.1%)	9 (36%)	185 (57.6%)	39 (47.6%)	0.046
Secondary progressive course (SP), *n* (%)	15 (28.3%)	11 (44%)	92 (28.7%)	32 (39.1%)	
Primary progressive course (PP), *n* (%)	12 (22.6%)	5 (20%)	43 (13.4%)	11 (13.4%)	
Clinically isolated syndrome (CIS), *n* (%)	0 (0%)	0 (0%)	1 (0.3%)	0 (0%)	

**Table 2:** MSWS-12/IT.

	Nelle precedenti 2 settimane quanto la SM ha: In the past two weeks, how much has your MS.	Not at all	Little	Fairly	Very	Extremely
1	Limitato la sua capacità di camminare Limited your ability to walk?	1	2	3	4	5

2	Limitato la sua capacità di correre Limited your ability to run?	1	2	3	4	5

3	Limitato la sua capacità di salire/scendere le scale Limited your ability to climb up and down stairs?	1	2	3	4	5

4	Reso più difficile stare in piedi mentre si svolge una attività Made standing when doing things more difficult?	1	2	3	4	5

5	Limitato la sua capacità di equilibrio mentre sta in piedi o cammina Limited your balance when standing or walking?	1	2	3	4	5

6	Limitato le distanze delle sue camminate Limited how far you are able to walk?	1	2	3	4	5

7	Aumentato lo sforzo necessario a camminare Increased the effort needed for you to walk?	1	2	3	4	5

8	Reso necessario a domicilio l'uso di supporti per camminare (bastone, appoggiarsi al muro) Made it necessary for you to use support when walking indoors (e.g., holding on to furniture, using a stick, etc.)?	1	2	3	4	5

9	Reso necessario all'esterno l'uso di supporti per camminare (bastone, appoggiarsi al muro) Made it necessary for you to use support when walking outdoors (e.g., using a stick, a frame, etc.)?	1	2	3	4	5

10	Rallentato la sua camminata Slowed down your walking?	1	2	3	4	5

11	Influenzato la fluidità della camminata Affected how smoothly you walk?	1	2	3	4	5

12	Reso necessario concentrarsi per camminare Made you concentrate on your walking?	1	2	3	4	5
